# Editorial: Reviews in transcatheter aortic valve implantation

**DOI:** 10.3389/fcvm.2025.1678985

**Published:** 2025-09-18

**Authors:** Mi Chen, Albert Markus Kasel

**Affiliations:** Department of Cardiology, University Hospital Zurich, Zurich, Switzerland

**Keywords:** transcatheter aoric valve implantation, balloon expandable valve, bicuspid aortic valve, pure aortic regurgitation, artificial intelligence

**Editorial on the Research Topic**
Reviews in Transcatheter Aortic Valve Implantation

## Introduction

Transcatheter Aortic Valve Implantation (TAVI) has evolved from a last-resort therapy for inoperable or high-risk elderly patients into a well-established treatment option across all surgical risk categories. Over the past two decades, TAVI has demonstrated remarkable clinical success and is now expanding its indications to include even asymptomatic aortic stenosis. As its use extends to younger and lower-risk populations, new challenges have emerged—ranging from lifetime management strategies such as redo-TAVI, to concerns about valve durability, anatomical complexities like bicuspid aortic valve (BAV), and the treatment of pure aortic regurgitation. In this editorial, we introduce the articles in this issue that collectively explore these evolving frontiers and outline future directions for the TAVI landscape.

### Anatomical challenges: bicuspid valves and complex anatomy

BAV has been considered an unfavorable anatomy for TAVI and was previously excluded from major randomized clinical trials. Key challenges associated with BAV include asymmetric and bulky calcification, large annulus and associated aortopathy (Chen et al.), and heterogeneous morphologies with variable raphe types. However, with the advent of new-generation transcatheter heart valve (THVs) and advances in implantation techniques (1–3), recent studies have reported comparable outcomes in BAV compared to tricuspid aortic valve (TAV) (4). Recently, a large cohort from China showed the first evidence suggesting that the type-0 BAV may be associated with better outcomes than both TAV and type-1 BAV (5). Paravalvular regurgitation (PVR) remains the most common complication associated with BAV, with more than 40% of patients exhibiting moderate or greater degrees of PVR (6). The calcified raphe causes not only PVR but also the horizontal shift of both balloon-expandable valves (BEVs) and self-expanding valves (SEVs). In type-1 BAV, where the raphe is typically located between the left and right coronary cusps, the horizontal shift tends to displace the THVs away from the coronary ostia, potentially offering a protective effect against coronary obstruction. However, the same displacement can lead to non- anatomic valve positioning, suboptimal hemodynamics, early prosthetic valve degeneration, and increased complexity in redo-TAVI procedures. These anatomical challenges underscore the need for heightened awareness and tailored strategies to ensure durable lifetime management in BAV patients undergoing TAVI.

### Pure aortic regurgitation

**Figure 1:**
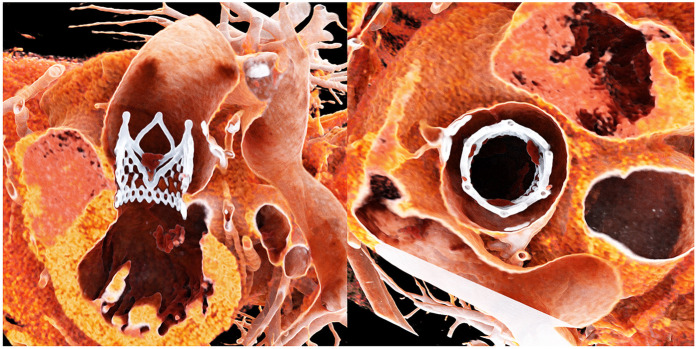
Title: post-procedural computed tomography of the jenaValve. Legend: Post-procedural CT demonstrates complete commissural alignment of the JenaValve with the native aortic cusps. The valve is also well positioned along the inflow–outflow axis, aligned with the native aortic anatomy.

Prior to the development of dedicated devices, several techniques using BEVs (7) and SEVs were employed to treat pure aortic regurgitation (PAR), with generally satisfactory results but a higher incidence of valve embolization. Gao et al. (https://www.frontiersin.org/journals/cardiovascular-medicine/articles/10.3389/fcvm.2025.1436789/full) reported a cohort of 552 patients treated with the J-valve system (JC Medical Inc., Burlingame, CA), a transapical device featuring three U-shaped anchors positioned at the nadirs of the native cusps to ensure stable sealing. The meta-analysis demonstrated a 96% procedure success rate. Another PAR-specific device, the JenaValve, has been approved in Europe for transfemoral approach. While the use of anchoring mechanisms improves sealing, it necessitates a larger sheath for delivery, slowing the transition from transapical to transfemoral approaches. Moreover, successful deployment requires precise alignment of the three anchors at the nadirs of each cusp, demanding rotational control of the delivery system, thereby increasing procedural complexity. However, this anchoring strategy allows for surgical-like valve placement with full commissural alignment, which may enhance long-term hemodynamics and prosthetic durability (Figure 1).

### Innovation at the forefront: new technologies in TAVI

The rapid evolution of TAVI has been driven by relentless technological innovation. Each new generation of transcatheter valves and delivery systems has aimed to tackle the shortcomings of its predecessors—whether by reducing complication rates, expanding patient eligibility, or simplifying the procedure. Key advancements and ongoing innovations include:

The latest fifth-generation Edwards SAPIEN 3 Ultra valve with RESILIA tissue exemplifies this progress, incorporating a bovine pericardial valve tissue treated with an advanced anti-calcification process (8). This proprietary RESILIA technology significantly slows calcium buildup on the leaflets—a major factor in structural valve degeneration—thereby aiming to extend valve lifespan and reduce the need for repeat interventions. In addition, the SAPIEN 3 Ultra RESILIA is the first transcatheter valve that can be stored dry (without liquid preservatives) thanks to its novel tissue preservation method, which simplifies handling and preparation during the procedure. By addressing the long-term durability challenges of earlier generations, such material innovations may broaden patient eligibility—even reaching younger, lower-risk patients who require valves with greater longevity. Moreover, new suture techniques on the commissures and the modified leaflet morphology allows significant improved hemodynamic with less gradients.

Another impressive innovation of BEVs is from the Siegel transcatheter heart valve system, notable for its 8 Fr delivery profile (New York Valves 2025). This Siegel valve employs a high-strength nickel-free and cobalt-free alloy frame with only 18 cells and is delivered through an ultra-low-profile sheath, enabling treatment of patients with small-caliber vessels. Early experience indicates that the device's precise deployment (with no foreshortening) and intrinsic commissural alignment allow for accurate positioning, which is expected to reduce conduction disturbances requiring pacemaker implantation. In initial cases, the Siegel valve achieved excellent haemodynamics with low transvalvular gradients and no paravalvular leak, outcomes that were scarcely imaginable with first-generation TAVI devices. Such breakthroughs illustrate the strides being made to refine TAVI therapy.

AI-driven tools now enable automatic segmentation and measurement of pre-TAVI CT scans, significantly improving efficiency and consistency in procedural planning. Virtual reality (VR) is also emerging as a valuable aid; in pilot studies, VR simulations accurately predicted paravalvular leak risk in bicuspid valves, highlighting its potential to enhance operator training and procedural outcomes.

TAVI continues to evolve through innovation, addressing increasingly complex anatomies and expanding toward younger, lower-risk populations. The advances highlighted in this issue underscore a future defined by precision, durability, and broader applicability.
